# Supplementary material for: Larval foraging decisions in competitive heterogeneous environments accommodate diets that support egg-to-adult development in a polyphagous fly
Source: R Soc Open Sci. 2019 Apr 17;6(4):190090. doi: 10.1098/rsos.190090 (PMC6502372; doi:10.1098/rsos.190090)
Supplement: Supplementary Material [file rsos190090supp1.pdf]

Supplementary Information: "Larval foraging decisions in competitive heterogeneous environments accommodate diets that support egg-to-adult development in a polyphagous fly"

Authors: Juliano Morimoto[1,2*], Shabnam Tarahi Tabrizi[1], Ida Lundback[1], Bishwo Mainali[1], Phillip W. Taylor[1], Fleur Ponton[1]

Author's affiliations:

1 - Department of Biological Sciences, Macquarie University, NSW 2109, Australia

2 - Programa de Pós-Graduação em Ecologia e Conservação, Federal University of Paraná, Curitiba, Brazil, 19031, CEP: 81531-990

*To whom correspondence should be addressed:

Juliano Morimoto

Address: Department of Biological Sciences, Macquarie University, NSW 2109, Australia

E-mail: juliano.morimoto@mq.edu.au

Supplementary Information

Supplementary tables

Table S1 – Diet recipes.

Ingredient	100%	80%	60%	40%	20%
**Brewer's Yeast (g)**	20.4	16.32	12.24	8.16	4.08
**Sugar (g)**	12.18	9.74	7.3	4.87	2.43
**Agar(g)**	1	1	1	1	1
**Citric Acid (g)**	2.3	2.3	2.3	2.3	2.3
**Nipagen (g)**	0.2	0.2	0.2	0.2	0.2
**Sodium benzoate (g)**	0.2	0.2	0.2	0.2	0.2
**Wheat Germ Oil (ml)**	0.2	0.2	0.2	0.2	0.2
**MiliQ Water (ml)**	100	100	100	100	100

Table S2 – Relative log-odds calculated from the multinomial logistic regression models.

Estimates showing the change in preference for each food option across time. Sample size: N = 15 foraging arenas.

	Larval density: 25 Relative Log-odds		Larval density: 50 Relative Log-odds		Larval density: 100 Relative Log-odds	
**Diet patch**	**Estimate**	**SE**	**Estimate**	**SE**	**Estimate**	**SE**
100%	0.063	0.395	0.200	0.298	0.435	0.258
80%	-0.245	0.400	-0.111	0.298	0.201	0.260
60%	-0.251	0.404	-0.070	0.305	0.222	0.262
40%	-0.490	0.456	-0.152	0.338	-0.367	0.301
20%	-5.511	75.829	-0.549	0.500	-5.686	0.185

Table S3 – Complete models for the effects of the diet treatment on pupal traits. Bold: p < 0.05.

Trait	Factor	df	Deviance	Residual df	Residual deviance	F-value	p-value
**Daily pupal recovery**	Replicate	1	0.002	52	4.006	0.353	0.555
	Diet treatment	2	0.036	50	3.970	3.818	**0.030**
	Day after egg collection	1	2.902	49	1.068	610.693	**<0.001**
	Day after egg collection^2	1	0.800	48	0.268	168.297	**<0.001**
	Diet treatment*Day after egg collection	2	0.047	46	0.221	4.994	**0.011**
	Diet treatment*Day after egg collection^2	2	0.012	44	0.209	1.232	0.301
**Pupal production**	Replicate	1	0.004	16	0.227	0.326	0.577
	Diet treatment	2	0.029	14	0.198	1.048	0.377
**Parental pupae weight**	Replicate	1	0.000	358	0.001	1.968	0.162
	Time	1	0.000	357	0.001	2.180	0.141
	Diet treatment	2	0.000	355	0.001	0.164	0.849
	Diet Treatment*Time	2	0.000	353	0.001	0.582	0.559

Table S4 – Complete models for the effects of the diet treatment on adult traits. Bold: p < 0.05.

Trait	Sex	Factor	df	Deviance	Residual df	Residual deviance	F-value	p-value
**Percentage of adult emergence**	-	Replicate	1	0.021	34	2.729	0.445	0.510
		Diet treatment	2	0.113	32	2.616	1.217	0.311
		Day of pupation	1	1.067	31	1.549	23.055	**<0.001**
		Diet treatment*Day of pupation	2	0.059	29	1.491	0.633	0.538
**Sex ratio**	-	Replicate	1	0.004	32	4.237	0.038	0.847
		Diet treatment	2	0.387	30	3.849	2.094	0.143
		Day of pupation	1	1.341	29	2.509	14.497	**<0.001**
		Diet treatment*Day of pupation	2	0.011	27	2.497	0.062	0.940
**Adult body mass**	***Male***	Replicate	1	0.33747	33	14.445	0.770	0.388
		Diet treatment	2	0.33309	31	14.112	0.380	0.687
		Day of pupation	1	0.01773	30	14.095	0.041	0.842
		Diet treatment*Day of pupation	2	1.82837	28	12.266	2.087	0.143
	Female	Replicate	1	1.0799	33	27.788	1.341	0.257
		Diet treatment	2	1.5192	31	26.269	0.943	0.401
		Day of pupation	1	0.0096	30	26.259	0.012	0.914
		Diet treatment*Day of pupation	2	3.708	28	22.552	2.302	0.119

Table S5 – Complete models for the effects of the diet treatment on adult cold tolerance. Bold: p < 0.05. Sample size: *N* = 48 individuals.

	**Cold tolerance**			
Analysis of Deviance	***Cox model***			
**Factor**	**loglik**	**Chisq**	**df**	**Pr(>|Chi|)**
Replicate	-138.02	5.310	1	**0.021**
Sex	-137.14	1.755	1	0.185
Diet Treatment	-135.72	2.839	2	0.242

Table S6 – Complete models for the effects of the diet treatment on adult reproduction and trans-generational effects. Bold: p < 0.05.

Trait	Factor	df	Deviance	Residual df	Residual deviance	F-value	p-value
**Fecundity per female**	Replicate	1	33.670	70	2619.50	1.440	0.235
	Number of males	1	8.840	69	2610.60	0.378	0.541
	Diet treatment	2	103.360	67	2507.30	2.210	0.118
	Age of the group	1	949.930	66	1557.30	40.625	**<0.001**
	Age of the group^2	1	89.510	65	1467.80	3.828	0.055
	Diet treatment*Age of the group	2	65.200	63	1402.60	1.394	0.256
	Diet treatment*Age of the group^2	2	0.980	61	1401.70	0.021	0.979
**Total fecundity per female**	Replicate	1	164.23	16	5687.5	0.458	0.510
	Diet treatment	2	1026.08	14	4661.4	1.430	0.272
**Average weight of offspring pupae**	Replicate	1	0.000	28	0.000	3.857	0.062
	Fecundity per female of the parental group	1	0.000	27	0.000	9.355	**0.006**
	Diet treatment	2	0.000	25	0.000	0.164	0.850
	Age of the parental group	1	0.000	24	0.000	1.703	0.205
	Diet treatment*Age of parental group	2	0.000	22	0.000	3.233	0.059

Table S7 – Complete models for the effects of the diet treatment on adult flight ability.

Trait	Factor	df	Deviance	Residual df	Residual deviance	F-value	p-value
**Average pupae weight (100 pupae)**	Replicate	1	0.000	16	0.000	2.901	0.111
	Diet treatment	2	0.000	14	0.000	0.085	0.919
**Percentage of adult emergence**	Replicate	1	0.022	16	1.605	0.266	0.614
	Diet treatment	2	0.458	14	1.147	2.803	0.095
**Sex ratio**	Replicate	1	0.010	16	0.795	0.196	0.665
	Diet treatment	2	0.108	14	0.687	1.096	0.361
**Percentage of partial emergence**	Replicate	1	4.8762	16	51.124	1.621	0.224
	Diet treatment	2	9	14	42.124	1.496	0.258
**Percentage of fliers**	Replicate	1	0.015	16	4.599	0.060	0.809
	Diet treatment	2	0.716	14	3.883	1.466	0.264
**Rate of fliers**	Replicate	1	0.008	16	2.426	0.064	0.805
	Diet treatment	2	0.298	14	2.128	1.197	0.331

Supplementary figures

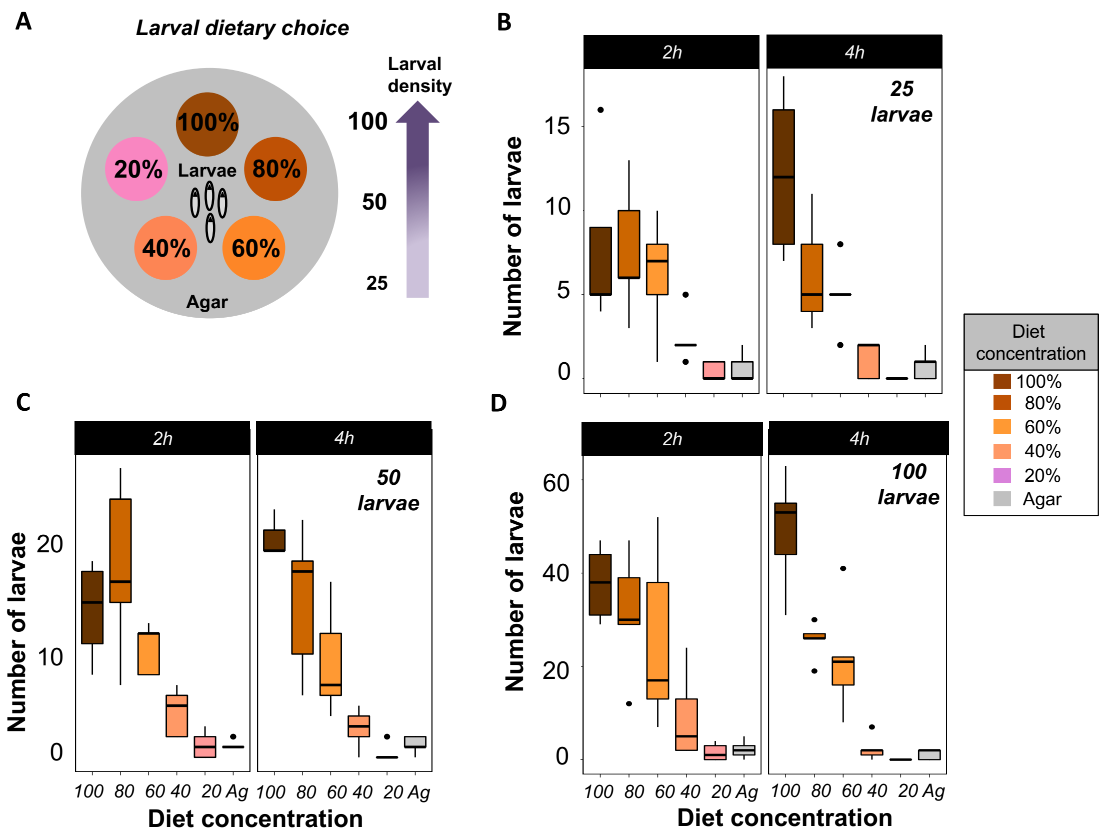

Figure S1 - Plots showing the number of larvae choosing each of the 5 diet treatments in the foraging arena. A) Schematic representation of our experimental setup for the larvae dietary choices. B-D) Number of larvae choosing each of the 5 diets in two different time points (2h and 4h) after larvae were placed in the arena. The density of individuals in the foraging group was 25 (B), 50 (C), and 100 (D) larvae.

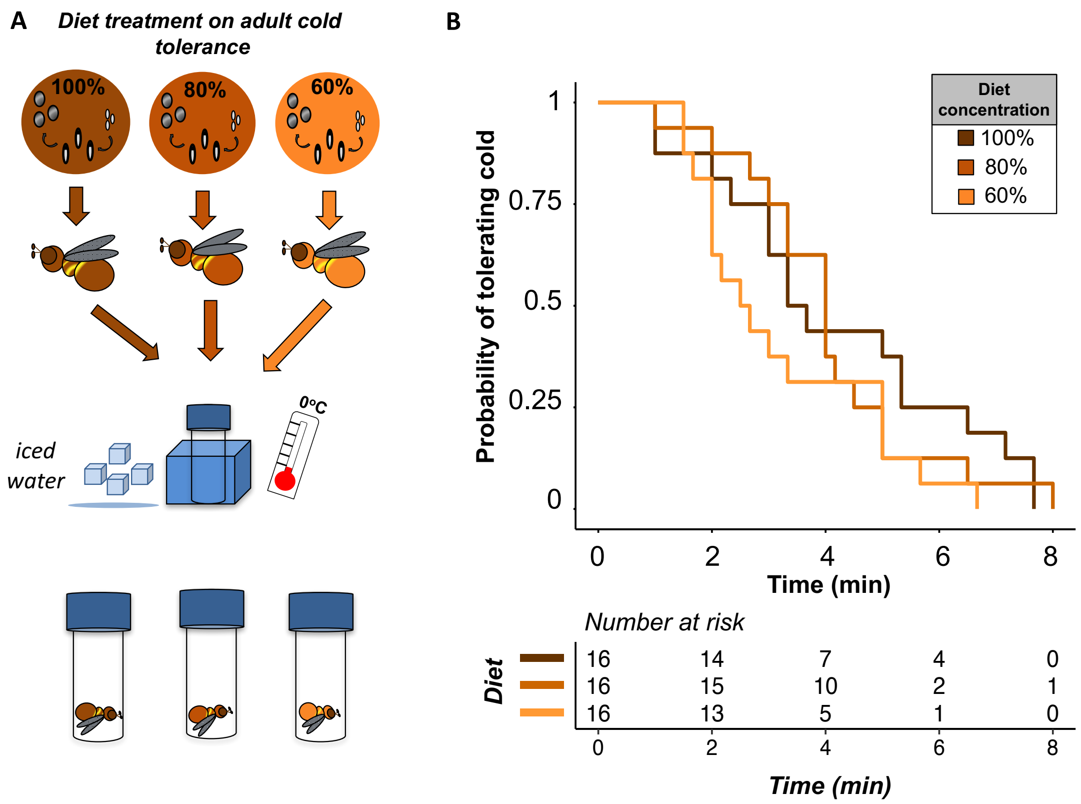

Figure S2 - The effects of diet treatment on adult cold tolerance. A) Schematic representation of our experimental setup for the cold tolerance experiment. B) Likelihood of tolerating cold at different time points throughout the duration of the cold tolerance experiment. Table shows the number of individuals at risk at different time points (in minutes).
